# Case Report: Paraganglioma in the sellar region: longitudinal observation and surgical outcome

**DOI:** 10.3389/fonc.2023.1090615

**Published:** 2023-05-23

**Authors:** Yingjie Wang, Xuan Yang, Qianquan Ma, Van Halm-Lutterodt Nicholas, Jianjun Sun, Xiaofang Zhao, Weihai Liu, Chenlong Yang

**Affiliations:** ^1^ Department of Neurosurgery, Peking University Third Hospital, Peking University, Beijing, China; ^2^ Center for Precision Neurosurgery and Oncology of Peking University Health Science Center, Peking University, Beijing, China; ^3^ Beijing Tongren Eye Center, Beijing Key Laboratory of Intraocular Tumor Diagnosis and Treatment, Beijing Ophthalmology & Visual Sciences Key Lab, Medical Artificial Intelligence Research and Verification Key Laboratory of the Ministry of Industry and Information Technology, Beijing Tongren Hospital, Capital Medical University, Beijing, China; ^4^ Department of Neurosurgery, Inspired Spine Health, Burnsville, MN, United States

**Keywords:** paraganglioma, sellar, extra-adrenal, surgical treatment, case report

## Abstract

**Background:**

Paraganglioma in the sellar region is an extremely rare entity, with a limited number of cases reported in the literature. Due to the paucity of clinical evidence, the diagnosis and treatment of paragangliomas in the sellar region remain challenging. Herein, we reported a case of sellar paraganglioma with parasellar and suprasellar extension. Particularly, the dynamic evolution of this benign tumor within a 7-year longitudinal observation was presented. Additionally, the relevant literature regarding sellar paraganglioma was comprehensively reviewed.

**Case description:**

A 70-year-old woman presented with progressive visual deterioration and headache. Brain magnetic resonance imaging demonstrated a mass in the sellar region with parasellar and suprasellar extension. The patient refused surgical treatment. Seven years later, brain magnetic resonance imaging showed the lesion significantly progressed. Neurological examination revealed bilateral tubular contraction of visual fields. Laboratory examinations showed endocrine hormone levels were normal. Surgical decompression was performed *via* a subfrontal approach, and subtotal resection was achieved. Histopathological examination confirmed a diagnosis of paraganglioma. Postoperatively, she developed hydrocephalus, and ventriculoperitoneal shunting was performed. Eight months later, cranial CT showed no recurrence of the residual tumor, and the hydrocephalus had been relieved.

**Conclusion:**

Paraganglioma occurring in the sellar region is rare, and the preoperative differential diagnosis is difficult. Owing to the infiltration to the cavernous sinus and internal carotid, complete surgical resection is usually impracticable. There has been no consensus regarding postoperative adjuvant radiochemotherapy for the tumor residue. *In-situ* recurrence and metastasis have been reported in the literature, and close follow-up is warranted.

## Introduction

Paragangliomas refer to benign neuroendocrine neoplasms originating from paraganglionic tissue, which are derived from neural crest progenitor cells. Except for the adrenal medulla, paragangliomas predominantly arise in the head and neck region, most commonly involving the carotid bodies, the jugular glomus, and the vagal bodies (1, 2). According to the literature, extra-adrenal paragangliomas account for only 10% to 15% of all paragangliomas (3); paragangliomas affecting the central nervous system are particularly rare, comprising approximately 0.6% of all head and neck neoplasms (4). Although paragangliomas are generally considered to be indolent entities corresponding histologically to World Health Organization (WHO) grade I (5), a high recurrence rate following surgical resection (10% for paragangliomas of carotid body and 50% to 60% for those occurring in other sites) and even metastasis (~10%) have been reported (6). Previously, we reported a consecutive surgical series of 19 patients with pathologically diagnosed spinal paragangliomas, in which we noted a considerable risk of *in-situ* recurrence Sellar paragangliomas are extremely unusual as paraganglia do not normally exist in this region, which has been only reported in very limited cases (7–36). This may be secondary to the persistence of paraganglionic tissue caused by deficient involution during early life. In this study, we presented a case of sellar paraganglioma with parasellar and suprasellar extension. Particularly, the dynamic evolution of this benign tumor within a 7-year longitudinal observation was presented. Additionally, the relevant literature regarding sellar paraganglioma was comprehensively reviewed.

## Case description

### History and clinicoradiological evaluations

**Figure 1:**
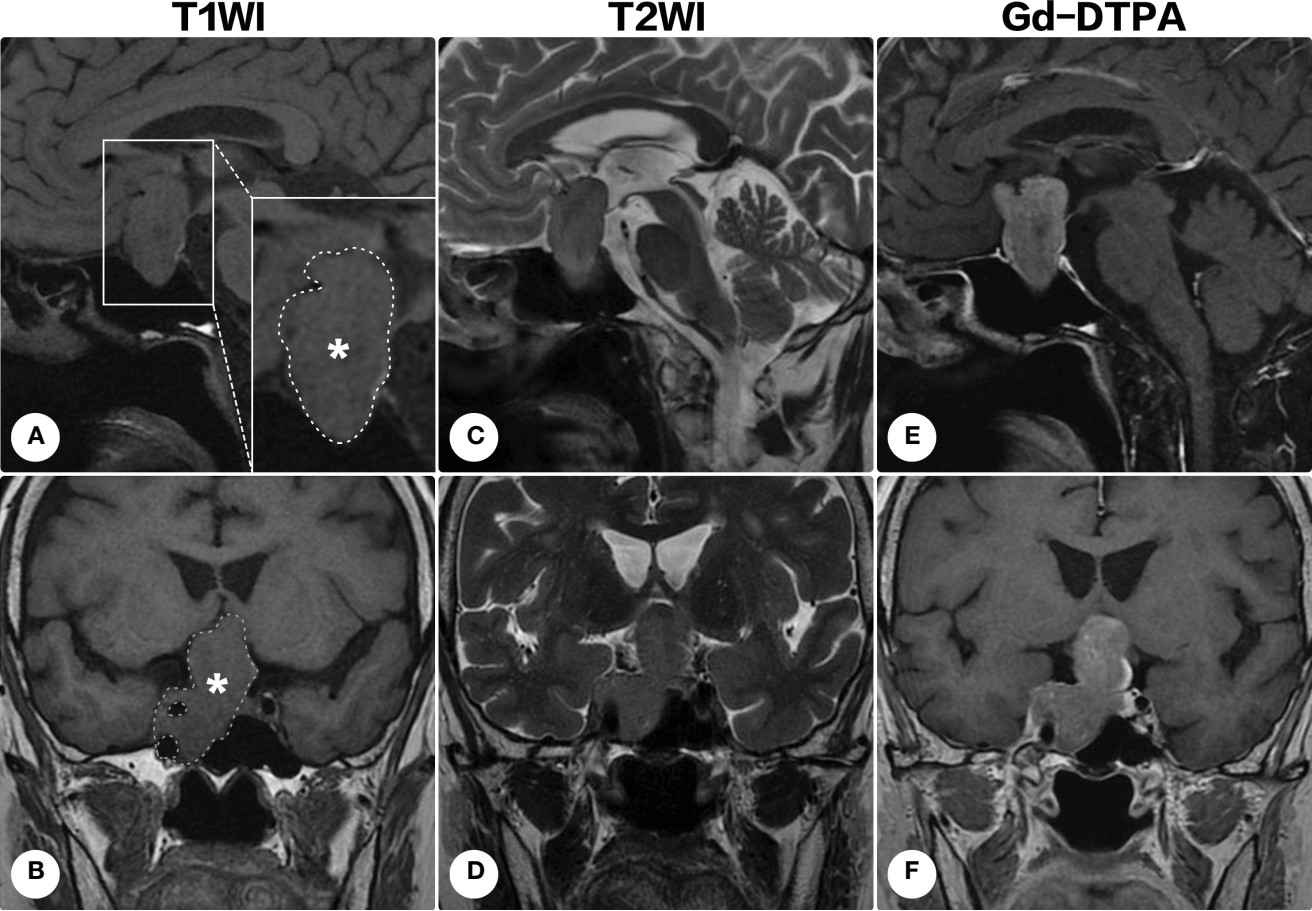
Brain magnetic resonance imaging on the first admission. Brain magnetic resonance imaging revealed a space-occupying lesion in the sellar region with right parasellar and suprasellar extension. The lesion (indicated by asterisks and outlined by dashed lines) appeared isointense on T1-weighted imaging [**(A)**, sagittal; **(B)** coronal] and heterogenous signals on T2-weighted imaging [**(C)**, sagittal; **(D)** coronal]. Contrast-enhanced T1-weighted images demonstrated a remarkable homogeneous enhancement [**(E)** sagittal; **(F)**, coronal].

A 70-year-old woman presented with a 3-year history of visual deterioration and headache. Physical examination revealed binocular severe visual disturbance. Brain magnetic resonance imaging (MRI) was requested, yielding a space-occupying lesion in the sellar region with right parasellar and suprasellar extension (**Figure 1**). The mass was irregular in shape displaying isointensity on T1-weighted imaging and heterogenous signals on T2-weighted imaging, and contrast enhancement was notable after the administration of gadolinium diethylenetriamine pentaacetic acid (Gd-DTPA). Surgical resection of the lesion *via* a subfrontal approach was recommended, but the patient refused it considering the advanced age and mild symptoms.

**Figure 2:**
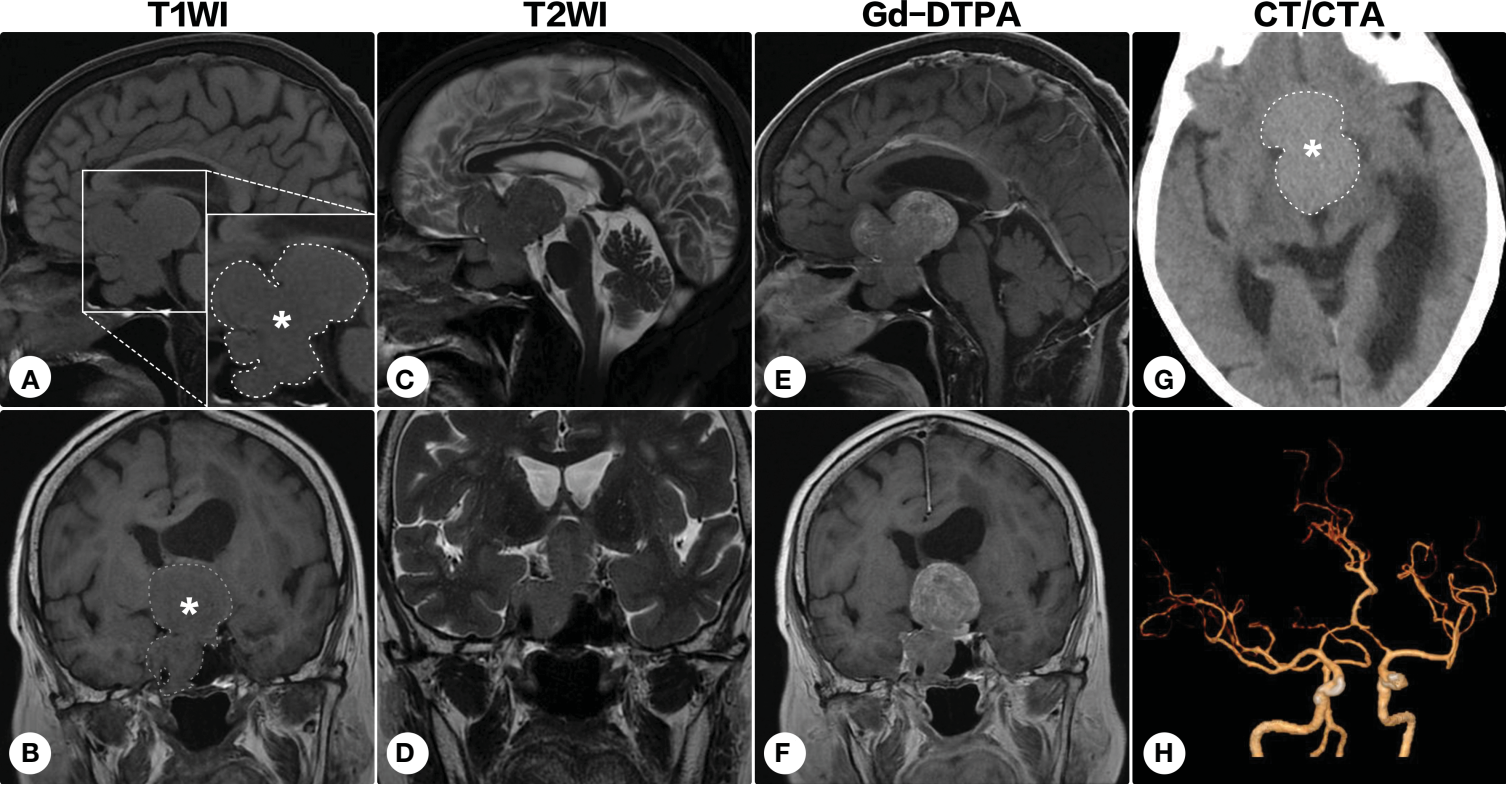
Brain magnetic resonance imaging after a 7-year conservative observation. Brain magnetic resonance imaging showed the lesion (indicated by asterisks and outlined by dashed lines) significantly progressed [**(A, C, E)** sagittal; **(B, D, F)** coronal]. On cranial computed tomography, the mass was hyperdense **(G)**. Computed tomographic angiography identified no significant vascular aberrance **(H)**.

**Figure 3:**
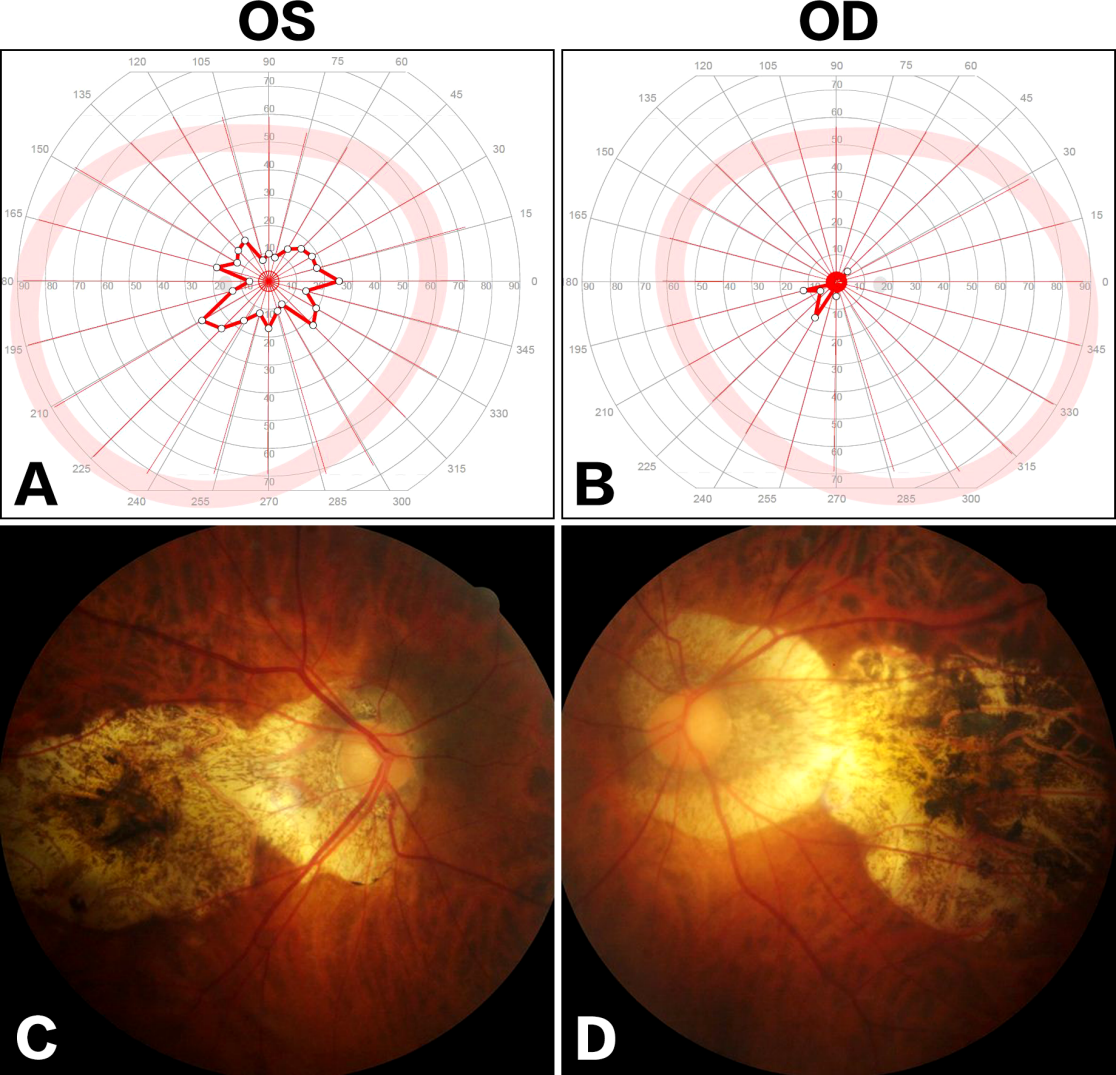
Ophthalmologic examinations. Visual field examination showed bilateral tubular contraction of visual fields [**(A)** OS; **(B)** OD]. Fundus examination demonstrated posterior staphyloma and tigroid fundus [**(C)**, OS; **(D)** OD].

During the following years, the visual field defect was progressively aggravated. The latest brain MRI after a 7-year conservative observation demonstrated the lesion significantly progressed (**Figure 2**), and the patient was readmitted. Ophthalmologic examination showed bilateral visual field defects and pathologic myopia with posterior staphyloma and tigroid fundus (**Figure 3**). Other cranial nerves were normal, and there were no sensorimotor dysfunctions. Laboratory examinations showed endocrine hormone levels (including prolactin [PRL], growth hormone [GH], follicle stimulating hormone [FSH], luteinizing hormone [LH], adrenocorticotropic hormone [ACTH], and thyroid hormones) were all within normal limits. Cranial computed tomography (CT) and MRI demonstrated a giant lobulated sellar mass with parasellar and suprasellar extension, and the optic chiasm was remarkably displaced; the lesion was isointense on T1-weighted imaging and heterogeneously hyperintense on T2-weighted imaging, with remarkable enhancement after administration of contrast medium (**Figures 2A-G**). CT angiography did not identify any significant vascular aberrance (**Figure 2H**).

### Surgical treatment

Preoperatively, a diagnosis of nonfunctional pituitary adenoma was suspected. As the main body of the lesion extended to the suprasellar and parasellar areas, the transnasal-sphenoidal approach may be difficult to achieve complete tumor resection. The patient underwent a surgical decompression *via* a subfrontal approach. Intraoperatively, the tumor was found to be rubbery with an abundant blood supply. The tumor tissue was closely attached to the internal carotid artery and cavernous sinus, and subtotal resection was eventually achieved.

### Histopathological examination

**Figure 4:**
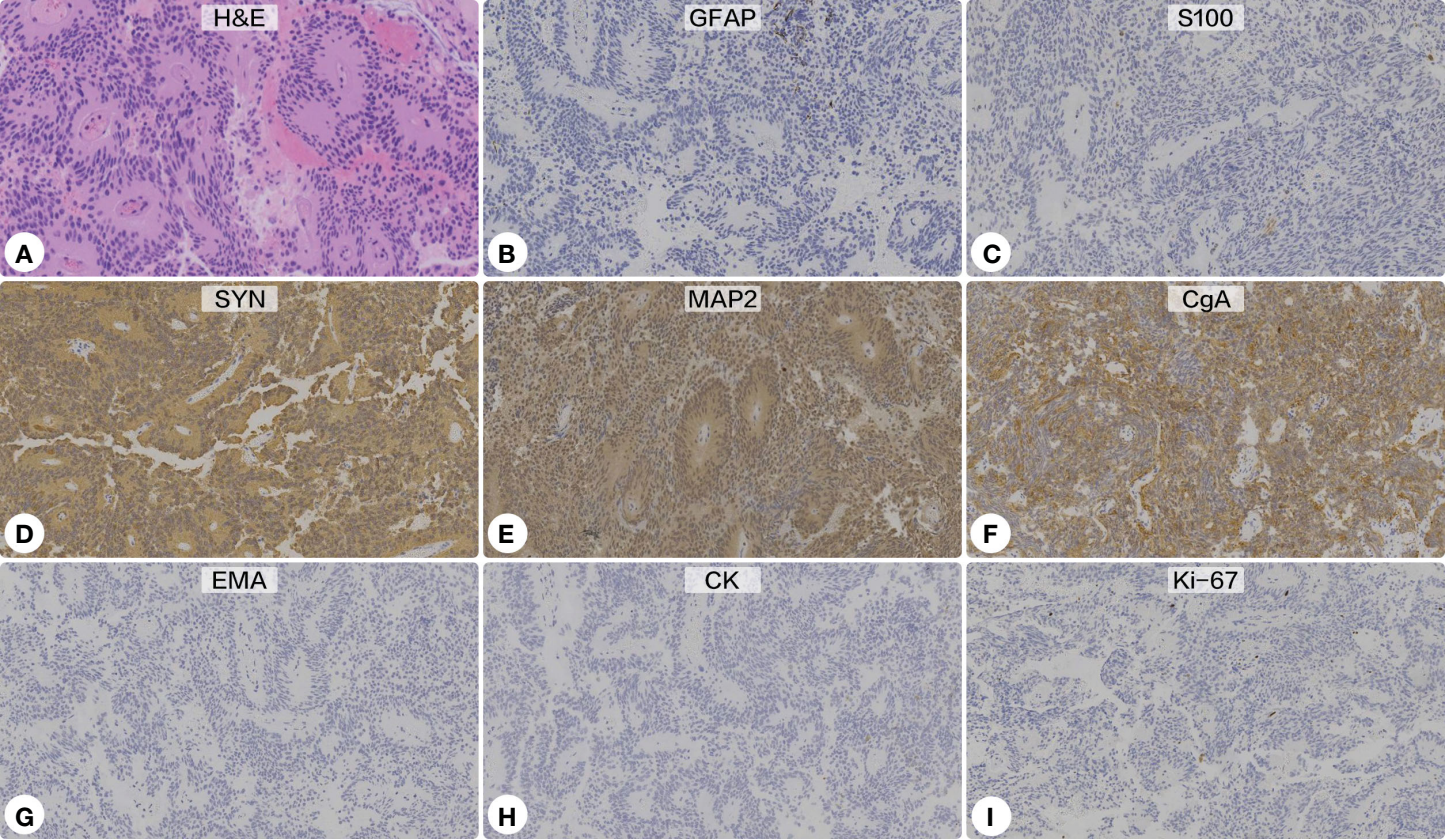
Histopathological and immunohistochemical examinations. **(A)** Hematoxylin-eosin staining showed a lobulated pattern and cellular nests surrounded by vascular fibrous septa, which were consistent with the morphological characteristics of paraganglioma (Zellballen pattern) (Original magnification ×200). Immunohistochemical stains showed the tumor cells were negative for glial fibrillary acidic protein **(B)** and S100 protein **(C)**, but strongly positive for synaptophysin **(D)**, microtubule-associated protein 2 **(E)**, and chromogranin A **(F)**. Additionally, the tumor showed no immunoreactivity against epithelial membrane antigen **(G)** or cytokeratin **(H)**. The Ki-67 proliferation index was <5% **(I)**.

Histopathological sections revealed a tumor with a lobulated pattern and cellular nests surrounded by vascular fibrous septa (**Figure 4**). The irregular and lobulated clusters of cuboidal cells were consistent with the morphological characteristics of paraganglioma (Zellballen pattern). Immunohistochemical stains showed the tumor cells were strongly positive for synaptophysin (SYN), chromogranin A (CgA), and microtubule-associated protein 2 (MAP-2), but negative for glial fibrillary acidic protein (GFAP), S100 protein, epithelial membrane antigen (EMA), cytokeratin (CK), CD68, and neuronal nuclear antigen (NeuN). Additionally, the tumor cells showed no immunoreactivity against pituitary cell-lineage transcription factors (T-PIT, PIT-1, SF-1), thyroid transcription factor 1 (TTF-1), or endocrine markers (PRL, GH, thyroid stimulating hormone [TSH], ACTH, FSH, LH). The Ki-67 proliferation index was <5%. A diagnosis of paraganglioma was made.

### Postoperative course

**Figure 5:**
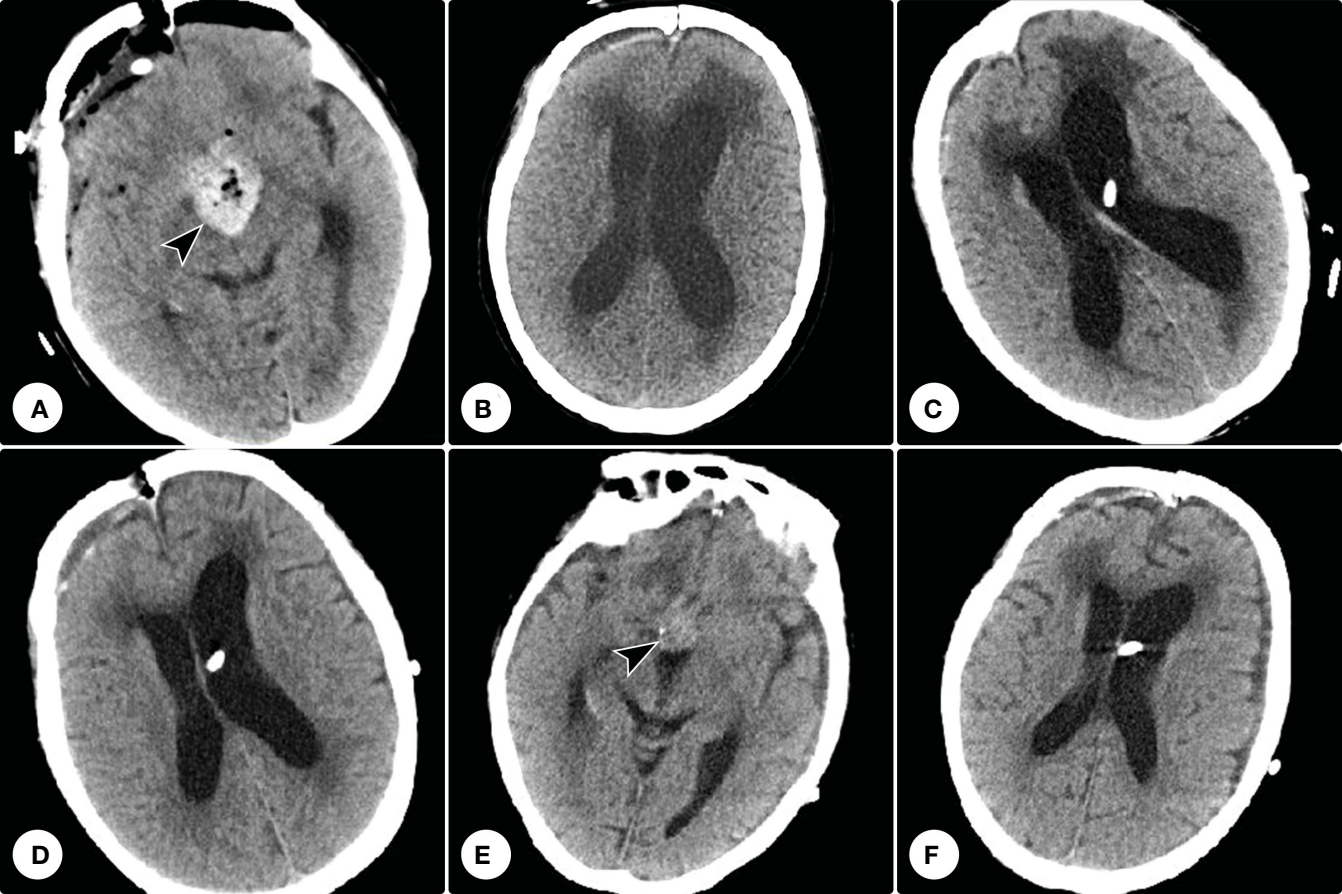
Postoperative and follow-up computed tomography examinations. **(A)** Postoperative computed tomography confirmed the tumor was subtotally removed, and the tumor cavity was filled with hemostatic material (arrowhead). **(B)** Repeated computed tomography showed ventricle dilation with interstitial edema. **(C)** Computed tomography showed a ventriculoperitoneal shunting was performed. **(D)** A week postoperatively, computed tomography showed the ventricles shrank. **(E, F)** Eight months postoperatively, cranial CT showed no recurrence of the tumor residue and the hemostatic material had been absorbed **(E)**, and the hydrocephalus had been relieved **(F)**.

Postoperative CT confirmed the tumor was subtotally removed, and the tumor cavity was filled with hemostatic material (**Figure 5A**). The patient’s visual function showed no significant improvement. One month after the operation, she developed gait disturbance and urinary incontinence. Repeated cranial CT showed ventricle dilation with interstitial edema (**Figure 5B**). A secondary hydrocephalus was diagnosed and a ventriculoperitoneal shunting was performed (**Figure 5C**). A week postoperatively, the neurological deficiencies were partially improved, and CT showed the ventricles shrank (**Figure 5D**). Eight months postoperatively, cranial CT showed no recurrence of the residual tumor, and the hydrocephalus had been relieved (**Figures 5E, F**). However, she got pneumonia two months later and succumbed to respiratory failure.

### Literature review

We conducted a literature review to identify articles on sellar paragangliomas published up to November 1, 2022. We performed a systematic search of the PubMed and Embase databases using the terms “paraganglioma” and “sellar”. A total of 30 publications were retrieved, and all of them are case reports.

There were 13 females and 22 males (including our current case), with a female-to-male ratio of 1:1.69. The average age was 46.9 ± 22.5 (range, 13~84 years). The most common onset symptoms were visual deficits (68.6%) and headache (65.7%). All the patients underwent surgical treatment, and no complete resection was described. Postoperative adjuvant radiation was administered for controlling the tumor residue in 45.7% of all cases (16 of 35 cases), with a dosage of 40~50 Gy (20~28 fractions). Follow-up data were available in 23 cases; after an average follow-up period of 21.5 ± 28.8 months, two patients experienced *in-situ* recurrence, and metastasis was noted in two other cases. Demographic and clinical profiles were summarized in **Table 1**. Radiological characteristics were presented in **Table 2**.

**Table 1:** Demographic and clinical features of paragangliomas in the sellar region.

Authors/year	Case No.	Gender	Age (years)	Onset Symptoms	Duration	Treatment	Follow-up period	Outcome
Ho et al./1982 (7)	1	Male	65	Headache and VD	N.A.	Surgery and RT	4 months	Stable
Steel et al./1993 (8)	2	Female	44	Headache	3 years	Surgery and RT	1 year	Stable
	3	Female	41	Headache, nausea and vomiting	N.A.	Surgery and RT (50Gy)	1 year	Stable
Scheithauer et al./1996 (9)	4	Male	14	Headache	3 months	Surgery and RT (45 Gy/28 fractions)	7 years	Recurrence
Noble et al./1997 (10)	5	Male	71	VD	N.A.	Surgery	N.A.	N.A.
	6	Male	14	headache	N.A.	Surgery	N.A.	N.A.
Mokry et al./1998 (11)	7	Female	76	VD	6 months	Surgery	N.A.	N.A.
Del Basso De Caro et al./ 1998 (12)	8	Male	84	MD	2 months	Surgery	1 year	Stable
Sambaziotis et al./1999 (13)	9	Male	54	VD	N.A.	Surgery	N.A.	N.A.
Salame et al./2001 (14)	10	Female	48	Headache and VD	N.A.	Surgery	N.A.	N.A.
Laquis et al./2001 (15)	11	Female	15	Headache, exophthalmos and VD	N.A.	Surgery and RT	N.A.	N.A.
Hertel et al./2003 (16)	12	Female	51	Headache and facioplegia	N.A.	Surgery and RT	3 months	Stable
Arkha et al./2003 (17)	13	Female	58	VD and exophthalmos	N.A.	Surgery	N.A.	N.A.
Riopel et al./2004 (18)	14	Male	66	Headache and VD	N.A.	Surgery	N.A.	N.A.
Zorlu et al./2005 (19)	15	Male	37	VD	1 year	Surgery	20 months	Tumor shrinkage
Naggara et al./2005 (20)	16	Male	47	MD and VD	N.A.	Surgery	N.A.	N.A.
Boari et al./2006 (21)	17	Male	52	Headache	N.A.	Surgery	3 years	Stable
Peltier et al./2007 (22)	18	Female	51	Headache, VD and oculomotor palsy	N.A.	Surgery and RT (45Gy)	N.A.	N.A.
Sinha et al./2008 (23)	19	Male	18	Headache and VD	2 months	Surgery and RT	4 months	Metastasis
Ozum et al./2008 (24)	20	Male	70	Headache	N.A.	Surgery and RT (50Gy)	N.A.	N.A.
Lu et al./2009 (25)	21	Male	81	Headache and VD	3 weeks	Surgery	4 months	Death
Haresh et al./2009 (26)	22	Male	17	Headache and VD	2 months	Surgery and RT (50 Gy/25 fractions)	4 months	Metastasis
Albert et al./2011 (27)	23	Male	63	Exophthalmos	5 years	Surgery and RT	6 weeks	Stable
Do Nascimento et al./2012 (28)	24	Female	33	Headache and VD	N.A.	Surgery	N.A.	N.A.
Chaudhry et al./2013 (29)	25	Male	44	Headache and VD	1 year	Surgery	20 months	Tumor shrinkage
Karlekar et al./2018 (30)	26	Male	19	VD	N.A.	Surgery and RT	1 year	Stable
Lyne et al./2019 (31)	27	Female	73	Headache and VD	N.A.	Surgery	6 months	Stable
Tan et al./2019 (32)	28	Female	40	VD and headache	1 month	Surgery and RT (50 Gy/28 fractions)	8 months	Recurrence
	29	Female	59	SIADH	N.A.	Surgery	3 months	Stable
Schueth et al./2020 (33)	30	Male	81	VD and MD	2 years	Surgery	2.5 years	Stable
Vasoya et al./2020 (34)	31	Male	13	Headache and VD	6 months	Surgery and RT (50 Gy,25 fractions)	4 years	Tumor shrinkage
	32	Male	20	VD	3 months	Surgery and RT (40 Gy,20 fractions)	8 months	Tumor shrinkage
Stojanoski et al./2021 (35)	33	Male	31	Headache and nausea	2 months	Surgery and RT (48 Gy,24 fractions)	10 years	Recurrence and metastasis
Ghaisas et al./2022 (36)	34	Male	20	Headache and VD	2 years	Surgery and RT (40 Gy/20 fractions)	3 years	Tumor shrinkage
Present Case	35	Female	70	Headache and VD	3 years	Surgery	8 months	Stable

VD, visual deficit; MD, mental disorder; SIADH, syndrome of inappropriate antidiuretic hormone secretion; N.A., not available; RT, radiation therapy.

**Table 2:** Radiological characteristics of paragangliomas in the sellar region.

Authors/year	Case No.	T1-weighted imaging	T2-weighted imaging	Gd-DTPA	Involvement				Bone Destruction
					Cavernous Sinus	Internal Carotid Artery	Optic Pathway	The Third Ventricle	
Ho et al./1982	1	N.A.	N.A.	N.A.	Right	N.A.	N.A.	N.A.	N.A.
Steel et al./1993	2	Isointense	N.A.	Enhancement	Left	Encasement	N.A.	N.A.	N.A.
	3	N.A.	N.A.	Enhancement	Bilateral	Encasement	N.A.	N.A.	N.A.
Scheithauer et al/1996	4	N.A.	N.A.	N.A.	N.A.	N.A.	N.A.	N.A.	N.A.
Noble et al./1997	5	Isointense	Hyperintense	Enhancement	Bilateral	Encasement	Yes	Yes	Yes
	6	N.A.	N.A.	Enhancement	Right	Encasement	Yes	No	N.A.
Mokry et al./1998	7	Isointense	Hyperintense	Enhancement	No	No	Yes	No	No
Del Basso De Caro et al./1998	8	N.A.	N.A.	N.A.	Left	N.A.	N.A.	Yes	N.A.
Sambaziotis et al./1999	9	N.A.	N.A.	N.A.	N.A.	N.A.	N.A.	N.A.	N.A.
Salame et al./2001	10	Isointense	Hyperintense	Enhancement	Left	Encasement	Yes	Yes	No
Laquis et al./2001	11	N.A.	N.A.	Enhancement	Bilateral	Encasement	Yes	No	Yes
Hertel et al./2003	12	Isointense	Hyperintense	Enhancement	Bilateral	Involvement	Yes	Yes	Yes
Arkha et al./2003	13	N.A.	N.A.	N.A.	N.A.	N.A.	N.A.	N.A.	N.A.
Riopel et al./2004	14	Isointense	N.A.	Enhancement	No	No	No	No	Yes
Zorlu et al./2005	15	N.A.	N.A.	Enhancement	N.A.	N.A.	N.A.	N.A.	N.A.
Naggara et al./2005	16	Isointense	Hyperintense	Enhancement	No	No	Yes	Yes	No
Boari et al./2006	17	N.A.	N.A.	Enhancement	No	No	Yes	No	No
Peltier et al./2007	18	N.A.	N.A.	Enhancement	Left	Encasement	N.A.	N.A.	N.A.
Sinha et al., 2008	19	N.A.	N.A.	Enhancement	N.A.	N.A.	No	No	No
Ozum et al./2008	20	N.A.	N.A.	N.A.	Bilateral	Encasement	No	No	Yes
Lu et al./2009	21	N.A.	Hyperintense	Enhancement	Right	Encasement	Yes	Yes	No
Haresh et al./2009	22	Isointense	Hyperintense	Enhancement	Bilateral	Encasement	Yes	Yes	No
Albert et al./2011	23	N.A.	Hyperintense	N.A.	Left	Encasement	N.A.	N.A.	N.A.
Do Nascimento et al./2012	24	N.A.	N.A.	Enhancement	Bilateral	Encasement	Yes	Yes	No
Chaudhry et al./2013	25	Isointense	Hyperintense	Enhancement	Bilateral	Encasement	Yes	No	Yes
Karlekar et al./2018	26	N.A.	N.A.	Enhancement	N.A.	N.A.	Yes	Yes	No
Lyne et al./2019	27	N.A.	N.A.	N.A.	Bilateral	Encasement	Yes	No	Yes
Tan et al./2019	28	N.A.	N.A.	Enhancement	Right	Encasement	Yes	No	No
	29	N.A.	N.A.	Enhancement	Bilateral	Encasement	Yes	No	No
Schueth et al./2020	30	N.A.	Hyperintense	N.A.	No	No	No	No	No
Vasoya et al./2020	31	N.A.	N.A.	Enhancement	Bilateral	Encasement	Yes	Yes	No
	32	N.A.	N.A.	Enhancement	Bilateral	Encasement	Yes	Yes	No
Stojanoski et al./2021	33	Isointense	Hyperintense	Enhancement	Bilateral	Encasement	No	No	No
Ghaisas et al./2022	34	N.A.	N.A.	Enhancement	N.A.	N.A.	Yes	Yes	No
Present Case	35	Isointense	Heterogeneous	Enhancement	Right	Encasement	Yes	Yes	No

N.A., not available.

## Discussion

Paragangliomas are relatively rare tumors that develop from the paraganglia of the autonomic nervous system. Because the sellar region lacks autonomic nervous tissue and there are no paraganglia, the derivation and etiology of sellar paragangliomas remain a mystery. Regarding its origin, there are two hypotheses: 1) the ‘embryonic remnants’ theory, proposing that paraganglioma may arise from the embryonic paraganglionic cells trapped around the pituitary; or 2) the ‘ectopic migration’ theory, holding that paraganglioma may originate from paraganglionic cells abnormally migrated across the tympanic or ciliary branches of the glossopharyngeal nerve to the cavernous sinus (8, 20). In this study, we found the majority of reported cases showed involvement of the cavernous sinus, which seems to support the latter view. Additionally, the most common onset symptom was visual decline rather than abnormal pituitary endocrine function, highly implying paraganglioma is an extra-pituitary lesion. In our current case, a longitudinal conservative observation demonstrated remarkable tumor growth. At the early stage, the tumor invaded the right cavernous sinus and encased the right internal carotid artery, while no osseous destruction or pituitary dysfunctions were noted. These clinicoradiological features prompted the cavernous sinus origin of sellar paragangliomas.

Preoperative diagnosis of sellar paragangliomas is extremely challenging, and differential diagnoses mainly include pituitary adenomas, meningiomas, craniopharyngiomas, germinomas, and Rathke cleft cysts. In most cases, sellar paragangliomas tend to be misdiagnosed as nonfunctional pituitary adenomas. The following potential characteristics may assist the diagnosis: 1) despite the giant size of the tumor, the patient’s endocrinal functions are usually normal; 2) just like the “salt and pepper” appearance on T2-weighted imaging that is frequently seen in peripheral paragangliomas, the high vascularization leads to heterogeneous intensity on T2-weighted imaging in sellar paragangliomas. Some scholars also proposed that functional imaging, such as ^18^F-DOPA positron emission tomography, may help confirm the neuroendocrine nature of tumors with high specificity (37, 38). Moreover, the imaging also shows additional advantages in diagnosing multifocality (co-existing paragangliomas in the rest of the body) and/or distant metastases (38, 39).

The most common symptom, visual deterioration, is caused by the tumor compression to the optic chiasma, and thus surgical resection of the tumor is the most effective treatment. The optional surgical strategies include the subfrontal approach, the transpterional approach, and transnasal-sphenoidal operations. In some cases, sellar paragangliomas are highly vascularized, and preoperative angiography may provide essential information for the identification of tumor blood supply. When a definitive supplying artery can be identified, preoperative endovascular embolization may significantly reduce the risk of intraoperative bleeding. In our case, the tumor was moderately enhanced after the administration of contrast medium and computed tomographic angiography showed no vascular aberrance, and thus endovascular embolization was not performed. Sellar paragangliomas are usually lobulated, and the cavernous sinus and internal carotid artery are always involved; therefore, intraoperative complete resection of the tumor may be extraordinarily difficult. For controlling the tumor residue, postoperative radiotherapy showed potential efficacies. Chemotherapy has not yet been reported for sellar paragangliomas.

The surgical outcomes of sellar paragangliomas varied distinctly. In most cases, surgery with or without radiation therapy leads to residual stability or even tumor shrinkage. Metastasis can also occur in a few cases, and close follow-up is indispensable.

## Conclusions

Paragangliomas occurring in the sellar region are rare, and preoperative differential diagnosis is difficult. Complete surgical resection of the tumor is usually impracticable, and postoperative adjuvant radiotherapy can be considered for the tumor residue. *In-situ* recurrence and metastasis have been reported in the literature, and close follow-up should be highlighted.

## Data availability statement

The original contributions presented in the study are included in the article/supplementary material. Further inquiries can be directed to the corresponding author.

## Ethics statement

Written informed consent was obtained from the individual legal guardian for the publication of any potentially identifiable images or data included in this article.

## Author contributions

Conception and design: CY. Acquisition of data: YW, XZ, WL, and QM. Analysis and interpretation of data: XY, QM, JS, and VHN. Drafting and critically revising the article: YW, XY, and CY. All authors contributed to the article and approved the submitted version.

## References

[1] Welander J Soderkvist P Gimm O. Genetics and clinical characteristics of hereditary pheochromocytomas and paragangliomas. Endocr Relat Cancer (2011) 18(6):R253–76. doi: 10.1530/ERC-11-0170 22041710

[2] Kliewer KE Cochran AJ. A review of the histology, ultrastructure, immunohistology, and molecular biology of extra-adrenal paragangliomas. Arch Pathol Lab Med (1989) 113(11):1209–18. 2684087

[3] Brahmbhatt P Patel P Saleem A Narayan R Young M. Retroperitoneal paraganglioma presenting as a chest pain: a case report. Case Rep Oncol Med (2013) 2013:329472. doi: 10.1155/2013/329472 23424694 PMC3572682

[4] Chang H Silva MA Torres AA Weng J de Lima Guido LP Velez-Torres J. A carcinoid tumor of the middle ear masquerading as a glomus tympanicum presenting with temporal lobe hemorrhage in a 70-year-old woman: case report and review of the literature. Neurochirurgie (2022) 68(6):654–60. doi: 10.1016/j.neuchi.2022.07.008 35905789

[5] Yang C Li G Fang J Wu L Yang T Deng X. Clinical characteristics and surgical outcomes of primary spinal paragangliomas. J Neurooncol (2015) 122(3):539–47. doi: 10.1007/s11060-015-1742-0 25720695

[6] Reithmeier T Gumprecht H Stolzle A Lumenta CB. Intracerebral paraganglioma. Acta Neurochir (Wien) (2000) 142(9):1063–6. doi: 10.1007/s007010070064 11086818

[7] Ho KC Meyer G Garancis J Hanna J. Chemodectoma involving the cavernous sinus and semilunar ganglion. Hum Pathol (1982) 13(10):942–3. doi: 10.1016/S0046-8177(82)80058-0 6290369

[8] Steel TR Dailey AT Born D Berger MS Mayberg MR. Paragangliomas of the sellar region: report of two cases. Neurosurgery (1993) 32(5):844–7. doi: 10.1227/00006123-199305000-00021 8492863

[9] Scheithauer BW Parameswaran A Burdick B. Intrasellar paraganglioma: report of a case in a sibship of von hippel-lindau disease. Neurosurgery (1996) 38(2):395–9. doi: 10.1097/00006123-199602000-00034 8869071

[10] Noble ER Smoker WR Ghatak NR. Atypical skull base paragangliomas. AJNR Am J Neuroradiol (1997) 18(5):986–90. PMC8338095 9159383

[11] Mokry M Kleinert R Clarici G Obermayer-Pietsch B. Primary paraganglioma simulating pituitary macroadenoma: a case report and review of the literature. Neuroradiology (1998) 40(4):233–7. doi: 10.1007/s002340050573 9592793

[12] Del Basso De Caro ML Siciliano A Cappabianca P Alfieri A de Divitiis E. Intrasellar paraganglioma with suprasellar extension: case report. Tumori (1998) 84(3):408–11. doi: 10.1177/030089169808400319 9678627

[13] Sambaziotis D Kontogeorgos G Kovacs K Horvath E Levedis A. Intrasellar paraganglioma presenting as nonfunctioning pituitary adenoma. Arch Pathol Lab Med (1999) 123(5):429–32. doi: 10.5858/1999-123-0429-IPPANP 10235503

[14] Salame K Ouaknine GE Yossipov J Rochkind S. Paraganglioma of the pituitary fossa: diagnosis and management. J Neurooncol (2001) 54(1):49–52. doi: 10.1023/A:1012535230135 11763422

[15] Laquis SJ Vick V Haik BG Fleming JC Wilson MW. Intracranial paraganglioma (glomus tumor) with orbital extension. Ophthalmic Plast Reconstr Surg (2001) 17(6):458–61. doi: 10.1097/00002341-200111000-00015 11766030

[16] Hertel F Bettag M Morsdorf M Feiden W. Paragangliomas of the parasellar region. Neurosurg Rev (2003) 26(3):210–4. doi: 10.1007/s10143-003-0266-9 12690532

[17] Arkha Y Boutarbouch M Lamalmi N Halefadl S Laamarti A Derraz S. Paraganglioma of the latero-sellar area. case report. Neurochirurgie (2003) 49(5):540–4. 14646820

[18] Riopel C Courville P Fabre B Callonnec F Bolognini B Marie JP. Parasellar paraganglioma: a case report. Ann Pathol (2004) 24(1):62–7. doi: 10.1016/S0242-6498(04)93903-X 15192541

[19] Zorlu F Selek U Ulger S Donmez T Erden E. Paraganglioma in sella. J Neurooncol (2005) 73(3):265–7. doi: 10.1007/s11060-004-5673-4 15980978

[20] Naggara O Varlet P Page P Oppenheim C Meder JF. Suprasellar paraganglioma: a case report and review of the literature. Neuroradiology (2005) 47(10):753–7. doi: 10.1007/s00234-005-1422-4 16047139

[21] Boari N Losa M Mortini P Snider S Terreni MR Giovanelli M. Intrasellar paraganglioma: a case report and review of the literature. Acta Neurochir (Wien) (2006) 148(12):1311–4. doi: 10.1007/s00701-006-0895-1 17039304

[22] Peltier J Fichten A Lefranc M Grunewald P Thelu F Toussaint P. Paraganglioma of the cavernous sinus. case report. Neurochirurgie (2007) 53(5):391–4. doi: 10.1016/j.neuchi.2007.06.006 17707867

[23] Sinha S Sharma MC Sharma BS. Malignant paraganglioma of the sellar region mimicking a pituitary macroadenoma. J Clin Neurosci (2008) 15(8):937–9. doi: 10.1016/j.jocn.2007.03.029 18482839

[24] Ozum U Egilmez R Yildirim A. Paraganglioma in pituitary fossa. Neuropathology (2008) 28(5):547–50. doi: 10.1111/j.1440-1789.2008.00885.x 18410271

[25] Lu JQ Khalil M Hu W Sutherland GR Clark AW. Tumor-to-tumor metastasis: esophageal carcinoma metastatic to an intracranial paraganglioma. J Neurosurg (2009) 110(4):744–8. doi: 10.3171/2008.9.JNS08397 19072308

[26] Haresh KP Prabhakar R Anand Rajan KD Sharma DN Julka PK Rath GK. A rare case of paraganglioma of the sella with bone metastases. Pituitary (2009) 12(3):276–9. doi: 10.1007/s11102-008-0099-1 18320326

[27] Albert A Ramirez JA Codere F Petrecca K. Sellar paraganglioma: a unique route to a rare destination case report and literature review. Clin Neurol Neurosurg (2011) 113(8):675–7. doi: 10.1016/j.clineuro.2011.04.002 21550714

[28] do Nascimento A Maranha LA Corredato RA Araujo JC Bleggi-Torres LF. 33 year-old woman with a large sellar tumor. Brain Pathol (2012) 22(6):869–70. doi: 10.1111/j.1750-3639.2012.00640.x PMC8029396 23050874

[29] Chaudhry NS Ahmad F Blieden C Morcos JJ. Suprasellar and sellar paraganglioma presenting as a nonfunctioning pituitary macroadenoma. J Clin Neurosci (2013) 20(11):1615–8. doi: 10.1016/j.jocn.2013.02.004 23876285

[30] Karlekar M Kumar S Jadhav S Lila A Bandgar T Shah N. Sellar paraganglioma. Clin Nucl Med (2018) 43(8):591–2. doi: 10.1097/RLU.0000000000002157 29894338

[31] Lyne SB Polster SP Fidai S Pytel P Yamini B. Primary sellar paraganglioma: case report with literature review and immunohistochemistry resource. World Neurosurg (2019) 125:32–6. doi: 10.1016/j.wneu.2019.01.094 30703592

[32] Tan CL Pang YH Lim KHC Sein L Codd PJ McLendon RE. Two extraordinary sellar neuronal tumors: literature review and comparison of clinicopathologic features. Am J Clin Pathol (2019) 151(3):241–54. doi: 10.1093/ajcp/aqy155 30551183

[33] Schueth EA Martinez DC Kulwin CG Bonnin JM Payner TD Ting JY. Recurrent primary intrasellar paraganglioma. Case Rep Otolaryngol (2020) 2020:2580160. doi: 10.1155/2020/2580160 32685227 PMC7336227

[34] Vasoya P Aryan S Thakar S Sivaraju L Ghosal N Hegde AS. Sellar-suprasellar paraganglioma: report of 2 cases and review of literature. World Neurosurg (2020) 140:293–300. doi: 10.1016/j.wneu.2020.04.157 32413561

[35] Stojanoski S Boldt HB Kozic D Patocs A Korbonits M Medic-Stojanoska M. Case report: malignant primary sellar paraganglioma with unusual genetic and imaging features. Front Oncol (2021) 11:739255. doi: 10.3389/fonc.2021.739255 34888235 PMC8650633

[36] Ghaisas S Rao KS Preethi A Rani PK. Suprasellar paraganglioma in a clinical setting of von hippel-lindau syndrome. BMJ Case Rep (2022) 15(3):e245907. doi: 10.1136/bcr-2021-245907 PMC8943740 35321910

[37] Taieb D Kaliski A Boedeker CC Martucci V Fojo T Adler JR Jr.. Current approaches and recent developments in the management of head and neck paragangliomas. Endocr Rev (2014) 35(5):795–819. doi: 10.1210/er.2014-1026 25033281 PMC4167435

[38] Timmers HJ Taieb D Pacak K. Current and future anatomical and functional imaging approaches to pheochromocytoma and paraganglioma. Horm Metab Res (2012) 44(5):367–72. doi: 10.1055/s-0031-1299712 PMC4714588 22399235

[39] Archier A Varoquaux A Garrigue P Montava M Guerin C Gabriel S. Prospective comparison of (68)Ga-DOTATATE and (18)F-FDOPA PET/CT in patients with various pheochromocytomas and paragangliomas with emphasis on sporadic cases. Eur J Nucl Med Mol Imaging (2016) 43(7):1248–57. doi: 10.1007/s00259-015-3268-2 26637204

